# Advances in Cardiovascular Pharmacology in Atherosclerotic-Related Therapeutic Areas: Addressing Patients’ Clinical Needs

**DOI:** 10.3390/jcm12113665

**Published:** 2023-05-25

**Authors:** Muntaser Omari, Mohammad Alkhalil

**Affiliations:** 1 Cardiothoracic Centre, Freeman Hospital, Newcastle-upon-Tyne NE7 7DN, UK; muntaser.omari@nhs.net; 2 Translational and Clinical Research Institute, Newcastle University, Newcastle-upon-Tyne NE2 4HH, UK

Over the last three decades, a significant improvement has been achieved in reducing cardiovascular morbidity and mortality [1]. Nonetheless, one in five patients returns with a second event within 5 years despite being on the guideline-recommended optimal medical therapy [2]. This risk is widely recognized to be heterogeneous, and characterizing the atherosclerosis process would allow tailored therapy and precise intervention at an individual level, aiming to reduce cardiovascular residual risk. Recent advances in pharmacotherapy have enabled physicians to target the main atherosclerotic-related processes, namely lipid, thrombosis, inflammation, and subsequent heart failure [3,4].

Low-density lipoprotein cholesterol (LDL-c) is a direct cause in the development of atherosclerotic cardiovascular disease (ASCVD) [5]. Numerous processes are implicated in early atherogenesis, including endothelial dysfunction, shear-stress-related events, platelet activation and aggregation, lipoprotein oxidation, inflammatory cell chemotaxis, the formation of foam cells, and smooth muscle migration [6]. Importantly, the retention of apo-lipoprotein B particles is a key determinant for the initiation and propagation of atherosclerotic plaque [6]. Statins and, subsequently, other LDL-c-lowering treatments, such as ezetimibe, proprotein convertase subtilisin/kexin (PCSK) 9 inhibitors, and bempedoic acid, have demonstrated that lowering LDL-c results in improving clinical outcomes, irrespective of the mechanism of the drug used [2,7,8,9]. Whilst the magnitude of LDL-c reduction was associated with a decrease in cardiovascular risk, a legacy effect was also evident underscoring the benefits of early initiation and long-term exposure to low LDL-c [10].

On the other hand, high-density lipoprotein (HDL)-c-raising therapies did not improve patients’ clinical outcomes [11,12,13,14] This is despite evidence from epidemiological studies supporting the role of HDL-c in the development of atherosclerosis [15,16]. Beyond its cargo of cholesterol, HDL particles have other functions, such as efflux capacity, that may have a protective role when managing patients with ASCVD [17]. Future studies will provide further insights on whether cholesterol efflux capacity could be a potential therapeutic target for patients with ASCVD.

Data from cohort-based and Mendelian randomization studies have highlighted an association between elevated triglycerides and worse clinical outcomes [18,19,20,21]. However, fibrates, niacin, and, more recently, marine-derived omega-3 fatty acids were not consistent in demonstrating a reduction in cardiovascular adverse events in response to the hypotriglyceridemic effect [22,23,24,25]. Triglycerides are not atherogenic per se, but are transported on lipoprotein particles that are increasingly recognized to be associated with the development of atherosclerotic plaque [21,26]. Whilst lowering triglycerides was associated with a reduction in future cardiovascular events, this effect was only modest and likely derived from the clearance of atherogenic lipoprotein particles [27]. 

The formation of platelet-rich thrombi is the principal cause of coronary obstruction in segments with atherosclerotic disease. This is why antiplatelet drugs are the cornerstone in the treatment of cardiovascular diseases [28,29]. Aspirin was the first approved antiplatelet medication targeting the cyclooxygenase-1 pathway. It achieves maximum platelet inhibition within 2 h following a loading dose and inhibits aggregation in 50% of circulating platelets for at least five days [30]. P_2_Y_12_ receptor is the second target to inhibit platelet activation, and developing safe therapies to antagonize this receptor has been a key step in reducing thrombotic sequalae of acute atherosclerotic plaque rupture [31]. Potent P_2_Y_12_ antagonists, prasugrel and ticagrelor, have led to a reduction in ischemic events, but at the expense of increasing bleeding risk when compared with clopidogrel [32,33]. Although these drugs provide faster and more consistent platelet inhibition and reduce high-platelet reactivity when compared with clopidogrel [34], a significant proportion of patients remained with high-platelet reactivity, particularly those with diabetes or post-acute coronary syndrome [35]. Additionally, patients presenting with acute myocardial infarction are susceptible to a delayed onset of platelet inhibition related to poor absorption or the use of opioids that can cause further delays in antiplatelet effects [36,37]. Nonoral P_2_Y_12_ antagonists have been proposed to bridge this gap and provide optimal platelet inhibition. Both intravenous (cangrelor) and subcutaneous (selatogrel) agents produce a rapid onset of platelet inhibition [38,39]. However, their roles in day-to-day clinical practice are yet to be determined. 

Targeting other platelet receptors, particularly glycoprotein (GP)_IIb/IIIa_, has also been assessed in large randomized clinical trials. The abundance of GP_IIb/IIIa_ receptor on the platelet surface and its direct involvement in platelet signaling and binding with fibrinogen have made it an excellent target for platelet inhibition [40]. In fact, patients who received intracoronary tirofiban, a reversible inhibitor of GP_IIbIIIa_, sustained a smaller infarct size when compared to placebo [41]. However, major bleeding was the main caveat to the routine use of GP_IIbIIIa_ inhibitor [42]. Protease-activated receptors (PAR) have emerged as another potential target to inhibit thrombin-mediated platelet activation [43]. Vorapaxar is an oral PAR1 antagonist that showed a significant reduction in cardiovascular events following ACS when compared to a placebo [44]. However, it was associated with an increased risk of bleeding, including intracranial bleeding. Different pharmacodynamics using slow and sustained platelet inhibition by targeting the PAR4 receptor may be a promising strategy of reducing platelet activation without increasing bleeding risk [45]. Clinical outcome data are anticipated which may support this hypothesis. 

Targeting fibrin formation using factor Xa or XIa inhibitors was proposed to attenuate thrombosis without increasing bleeding risks. Unlike Xa, XIa only resides in the intrinsic pathway and could provide the right balance by reducing thrombotic risk without disrupting hemostasis [46]. Numerous Phase II studies have reported the safety of factor Xia inhibitor, with ongoing Phase III trials establishing its efficacy. 

It has long been established that atherosclerosis is an inflammatory disease [3]. Immune cells, noncellular components such as interleukins and circulating microparticles, and, recently, the perivascular adipose tissue all contribute to this inflammatory role [47,48,49,50]. Aspirin and statins showed anti-inflammatory properties, quantified using highly sensitive (hs)-C-reactive protein, independently of their antiplatelet and cholesterol reduction properties, respectively [51,52].

The recent CANTOS (Canakinumab Anti-Inflammatory Thrombosis Outcomes Study) provided the most compelling evidence to date on the inflammatory hypothesis of atherothrombosis by reducing the risk of cardiovascular death, nonfatal myocardial infarction, and nonfatal stroke by 15% in patients with previous myocardial infarction and a hs-CRP ≥ 2 mg/L [53]. This landmark study highlighted the dual diagnostic and therapeutic benefits of detecting and targeting high levels of residual systemic inflammation in secondary cardiovascular prevention [53].

Subsequently, colchicine showed incremental benefits when added to standard treatment in patients with stable and unstable coronary presentation [54,55]. Intriguingly, its benefits were more evident in patients with diabetes, with almost twice the absolute risk reduction compared with nondiabetic patients, highlighting the inflammatory nature of the disease [56,57]. Other emerging anti-inflammatory treatments targeting the interleukin-6 pathway may provide additional benefits in patients with ASCVD [58].

Collectively, the residual atherosclerotic risk is widely heterogeneous. Attention should be sharply focused on identifying patients who may benefit maximally from novel treatment. Such treatments may be too expensive or associated with significant side effects that could offset any potential benefits when administered unselectively to all populations [59]. For example, 12 months of dual-antiplatelet treatment using potent P_2_Y_12_ antagonists remains the standard treatment in patients post-acute myocardial infarction. Extending the duration of dual-antiplatelet treatment provided additional ischemic benefits but with increased bleeding risks. Alternative strategies that could reduce bleeding risks and maintaining ischemic protection would be ideal for CAD patients. Three approaches have shown promising results to mitigate the bleeding risk. These include (1) early aspirin discontinuation [60], (2) de-escalating antiplatelet treatment either unselectively or guided by genotyping or platelet function [61], or (3) the use of scoring systems to balance bleeding and ischemic risks [62]. Importantly, these approaches have not been compared head-to-head and recommending one strategy over another remains challenging, notwithstanding the importance of certain clinical factors such as age and dialysis that should be considered in the decision-making process when using dual-antiplatelet therapy [63,64]. A similar approach could be utilized when subjecting patients to lipid-lowering or anti-inflammatory treatments. The use of plaque imaging would provide a direct evaluation of atherosclerotic plaque and provide important insights into its composition alongside its inflammatory status [49,50,65,66,67,68] Patients with a propensity to develop lipid-rich plaque may be candidates for intensive lipid-lowering treatment. Similarly, those with highly inflammatory plaque could be subjected to anti-inflammatory drugs. This model would be more cost-effective and reduce potential side effects compared to the unselective approach. An alternative strategy is to use high-risk clinical features, such as previous coronary artery bypass graft or polyvascular disease, to identify high-risk patients who may be eligible for-long term novel antiatherosclerotic treatments [56,69,70].

The heterogeneity of atherosclerotic disease features, alongside the variations in individuals’ responses to currently available therapies, dictates a more comprehensive approach to understand and quantify the subject’s residual risk. Therefore, characterizing atherosclerotic disease and matching its features to targeted therapies may offer an opportunity to achieve more accurate intervention, a step closer toward precision medicine.

## References

[1] Visseren F.L.J., Mach F., Smulders Y.M., Carballo D., Koskinas K.C., Back M., Benetos A., Biffi A., Boavida J.M., Capodanno D. (2021). ESC Guidelines on cardiovascular disease prevention in clinical practice. Eur. Heart J..

[2] Cannon C.P., Blazing M.A., Giugliano R.P., McCagg A., White J.A., Théroux P., Darius H., Lewis B.S., Ophuis T.O., Jukema J.W. (2015). Ezetimibe Added to Statin Therapy after Acute Coronary Syndromes. N. Engl. J. Med..

[3] Alkhalil M. (2021). Mechanistic Insights to Target Atherosclerosis Residual Risk. Curr. Probl. Cardiol..

[4] Alkhalil M., Choudhury R.P. (2018). Current concepts in atherosclerosis. Indian J. Thorac. Cardiovasc. Surg..

[5] Ference B.A., Ginsberg H.N., Graham I., Ray K.K., Packard C.J., Bruckert E., Hegele R.A., Krauss R.M., Raal F.J., Schunkert H. (2017). Low-density lipoproteins cause atherosclerotic cardiovascular disease. 1. Evidence from genetic, epidemiologic, and clinical studies. A consensus statement from the European Atherosclerosis Society Consensus Panel. Eur. Heart J..

[6] Williams K.J., Tabas I. (1995). The Response-to-Retention Hypothesis of Early Atherogenesis. Arter. Thromb. Vasc. Biol..

[7] Schwartz G.G., Steg P.G., Szarek M., Bhatt D.L., Bittner V.A., Diaz R., Edelberg J.M., Goodman S.G., Hanotin C., Harrington R.A. (2018). Alirocumab and Cardiovascular Outcomes after Acute Coronary Syndrome. N. Engl. J. Med..

[8] Sabatine M.S., Giugliano R.P., Keech A.C., Honarpour N., Wiviott S.D., Murphy S.A., Kuder J.F., Wang H., Liu T., Wasserman S.M. (2017). Evolocumab and Clinical Outcomes in Patients with Cardiovascular Disease. N. Engl. J. Med..

[9] Nissen S.E., Lincoff A.M., Brennan D., Ray K.K., Mason D., Kastelein J.J., Thompson P.D., Libby P., Cho L., Plutzky J. (2023). Bempedoic Acid and Cardiovascular Outcomes in Statin-Intolerant Patients. N. Engl. J. Med..

[10] O’donoghue M.L., Giugliano R.P., Wiviott S.D., Atar D., Keech A.C., Kuder J.F., Im K., Murphy S.A., Flores-Arredondo J.H., López J.A.G. (2022). Long-Term Evolocumab in Patients with Established Atherosclerotic Cardiovascular Disease. Circulation.

[11] Barter P.J., Caulfield M., Eriksson M., Grundy S.M., Kastelein J.J.P., Komajda M., Lopez-Sendon J., Mosca L., Tardif J.-C., Waters D.D. (2007). Effects of Torcetrapib in Patients at High Risk for Coronary Events. N. Engl. J. Med..

[12] Fayad Z.A., Mani V., Woodward M., Kallend D., Abt M., Burgess T., Fuster V., Ballantyne C.M., Stein E.A., Tardif J.-C. (2011). Safety and efficacy of dalcetrapib on atherosclerotic disease using novel non-invasive multimodality imaging (dal-PLAQUE): A randomised clinical trial. Lancet.

[13] Lincoff A.M., Nicholls S.J., Riesmeyer J.S., Barter P.J., Brewer H.B., Fox K.A.A., Gibson C.M., Granger C., Menon V., Montalescot G. (2017). Evacetrapib and Cardiovascular Outcomes in High-Risk Vascular Disease. N. Engl. J. Med..

[14] Bowman L., Hopewell J.C., Chen F., Wallendszus K., Stevens W., Collins R., Wiviott S.D., Cannon C.P., Braunwald E., Sammons E. (2017). Effects of Anacetrapib in Patients with Atherosclerotic Vascular Disease. N. Engl. J. Med..

[15] Miller G., Miller N. (1975). Plasma-High-Density-Lipoprotein Concentration and Development of Ischæmic Heart-Disease. Lancet.

[16] Di Angelantonio E., Sarwar N., Perry P., Kaptoge S., Ray K.K., Thompson A., Wood A.M., Lewington S., Sattar N., The Emerging Risk Factors Collaboration (2009). Major lipids, apolipoproteins, and risk of vascular disease. JAMA.

[17] von Eckardstein A., Nordestgaard B.G., Remaley A.T., Catapano A.L. (2023). High-density lipoprotein revisited: Biological functions and clinical relevance. Eur. Heart J..

[18] Sarwar N., Sandhu M.S., Ricketts S.L., Butterworth A.S., Di Angelantonio E., Boekholdt S.M., Ouwehand W., Watkins H., Triglyceride Coronary Disease Genetics Consortium, Emerging Risk Factors Collaboration (2010). Triglyceride-mediated pathways and coronary disease: Collaborative analysis of 101 studies. Lancet.

[19] Nordestgaard B.G., Benn M., Schnohr P., Tybjærg-Hansen A. (2007). Nonfasting Triglycerides and Risk of Myocardial Infarction, Ischemic Heart Disease, and Death in Men and Women. JAMA.

[20] Varbo A., Benn M., Tybjaerg-Hansen A., Nordestgaard B.G. (2013). Elevated remnant cholesterol causes both low-grade inflammation and ischemic heart disease, whereas elevated low-density lipoprotein cholesterol causes ischemic heart disease without inflammation. Circulation.

[21] Varbo A., Benn M., Tybjærg-Hansen A., Jørgensen A.B., Frikke-Schmidt R., Nordestgaard B.G. (2013). Remnant Cholesterol as a Causal Risk Factor for Ischemic Heart Disease. J. Am. Coll. Cardiol..

[22] Das Pradhan A., Glynn R.J., Fruchart J.C., MacFadyen J.G., Zaharris E.S., Everett B.M., Campbell S.E., Oshima R., Amarenco P., Blom D.J. (2022). Triglyceride Lowering with Pemafibrate to Reduce Cardiovascular Risk. N. Engl. J. Med..

[23] Landray M.J., Haynes R., Hopewell J.C., Parish S., Aung T., Tomson J., Wallendszus K., Craig M., Jiang L., HPS2-Thrive Collaborative Group (2014). Effects of extended-release niacin with laropiprant in high-risk patients. N. Engl. J. Med..

[24] Nicholls S.J., Lincoff A.M., Garcia M., Bash D., Ballantyne C.M., Barter P.J., Davidson M.H., Kastelein J.J.P., Koenig W., McGuire D.K. (2020). Effect of High-Dose Omega-3 Fatty Acids vs Corn Oil on Major Adverse Cardiovascular Events in Patients at High Cardiovascular Risk: The STRENGTH Randomized Clinical Trial. JAMA.

[25] Bhatt D.L., Steg P.G., Miller M., Brinton E.A., Jacobson T.A., Ketchum S.B., Doyle R.T., Juliano R.A., Jiao L., Granowitz C. (2019). Cardiovascular Risk Reduction with Icosapent Ethyl for Hypertriglyceridemia. N. Engl. J. Med..

[26] Wadstrom B.N., Pedersen K.M., Wulff A.B., Nordestgaard B.G. (2023). Elevated remnant cholesterol, plasma triglycerides, and cardiovascular and non-cardiovascular mortality. Eur. Heart J..

[27] Marston N.A., Giugliano R.P., Im K., Silverman M.G., O’donoghue M.L., Wiviott S.D., Ference B.A., Sabatine M.S. (2019). Association Between Triglyceride Lowering and Reduction of Cardiovascular Risk Across Multiple Lipid-Lowering Therapeutic Classes: A Systematic Review and Meta-Regression Analysis of Randomized Controlled Trials. Circulation.

[28] Alkhalil M., Džavík V., Bhatt D.L., Mehran R., Mehta S.R. (2022). Antiplatelet Therapy in Patients Undergoing Elective Percutaneous Coronary Intervention. Curr. Cardiol. Rep..

[29] Alkhalil M., Kuzemczak M., Bell A., Stern S., Welsford M., Cantor W.J., Goodman S.G. (2022). A practical approach to prescribing antiplatelet therapy in patients with acute coronary syndromes. Can. Med. Assoc. J..

[30] Layne K., Ferro A. (2017). Antiplatelet Therapy in Acute Coronary Syndrome. Eur. Cardiol..

[31] Mehta S.R., Yusuf S., Peters R.J., Bertrand M.E., Lewis B.S., Natarajan M.K., Malmberg K., Rupprecht H., Zhao F., Chrolavicius S. (2001). Unstable angina to prevent Recurrent Events trial I. Effects of pretreatment with clopidogrel and aspirin followed by long-term therapy in patients undergoing percutaneous coronary intervention: The PCI-CURE study. Lancet.

[32] Wallentin L., Becker R.C., Budaj A., Cannon C.P., Emanuelsson H., Held C., Horrow J., Husted S., James S., Katus H. (2009). Ticagrelor versus Clopidogrel in Patients with Acute Coronary Syndromes. N. Engl. J. Med..

[33] Wiviott S.D., Braunwald E., McCabe C.H., Montalescot G., Ruzyllo W., Gottlieb S., Neumann F.-J., Ardissino D., De Servi S., Murphy S.A. (2007). Prasugrel versus Clopidogrel in Patients with Acute Coronary Syndromes. N. Engl. J. Med..

[34] Thomas M.R., Storey R.F. (2016). Clinical significance of residual platelet reactivity in patients treated with platelet P2Y12 inhibitors. Vasc. Pharmacol..

[35] Winter M.-P., Grove E.L., De Caterina R., Gorog D.A., Ahrens I., Geisler T., Gurbel P.A., Tantry U., Navarese E., Siller-Matula J.M. (2017). Advocating cardiovascular precision medicine with P2Y12 receptor inhibitors. Eur. Heart J. Cardiovasc. Pharmacother..

[36] Alexopoulos D., Xanthopoulou I., Gkizas V., Kassimis G., Theodoropoulos K.C., Makris G., Koutsogiannis N., Damelou A., Tsigkas G., Davlouros P. (2012). Randomized Assessment of Ticagrelor Versus Prasugrel Antiplatelet Effects in Patients with ST-Segment–Elevation Myocardial Infarction. Circ. Cardiovasc. Interv..

[37] Silvain J., Storey R.F., Cayla G., Esteve J.B., Dillinger J.G., Rousseau H., Tsatsaris A., Baradat C., Salhi N., Hamm C.W. (2016). P2Y12 receptor inhibition and effect of morphine in patients undergoing primary PCI for ST-segment elevation myocardial infarction. Priv. Atl. Study Thromb Haemost..

[38] Bhatt D.L., Stone G.W., Mahaffey K.W., Gibson C.M., Steg P.G., Hamm C.W., Price M.J., Leonardi S., Gallup D., Bramucci E. (2013). Effect of Platelet Inhibition with Cangrelor during PCI on Ischemic Events. N. Engl. J. Med..

[39] Sinnaeve P., Fahrni G., Schelfaut D., Spirito A., Mueller C., Frenoux J.-M., Hmissi A., Bernaud C., Ufer M., Moccetti T. (2020). Subcutaneous Selatogrel Inhibits Platelet Aggregation in Patients with Acute Myocardial Infarction. J. Am. Coll. Cardiol..

[40] Li Z., Delaney M.K., O’Brien K.A., Du X. (2010). Signaling During Platelet Adhesion and Activation. Arter. Thromb. Vasc. Biol..

[41] Stone G.W., Maehara A., Witzenbichler B., Godlewski J., Parise H., Dambrink J.-H.E., Ochala A., Carlton T.W., Cristea E., Wolff S.D. (2012). Intracoronary abciximab and aspiration thrombectomy in patients with large anterior myocardial infarction: The INFUSE-AMI randomized trial. JAMA.

[42] De Luca G., Navarese E.P., Cassetti E., Verdoia M., Suryapranata H. (2011). Meta-Analysis of Randomized Trials of Glycoprotein IIb/IIIa Inhibitors in High-Risk Acute Coronary Syndromes Patients Undergoing Invasive Strategy. Am. J. Cardiol..

[43] Brass L.F. (2003). Thrombin and platelet activation. Chest.

[44] Tricoci P., Lokhnygina Y., Huang Z., Van de Werf F., Cornel J.H., Chen E., Wallentin L., Held C., Aylward P.E., Moliterno D.J. (2014). Vorapaxar with or without clopidogrel after non–ST-segment elevation acute coronary syndromes: Results from the Thrombin Receptor Antagonist for Clinical Event Reduction in Acute Coronary Syndrome trial. Am. Heart J..

[45] Wilson S.J., Ismat F.A., Wang Z., Cerra M., Narayan H., Raftis J., Gray T.J., Connell S., Garonzik S., Ma X. (2018). PAR4 (Protease-Activated Receptor 4) Antagonism With BMS-986120 Inhibits Human Ex Vivo Thrombus Formation. Arter. Thromb. Vasc. Biol..

[46] Weitz J.I., Eikelboom J.W. (2022). What Is the Future of Factor XI Inhibitors?. Circulation.

[47] Alkhalil M., Kearney A., Hegarty M., Stewart C., Devlin P., Owens C.G., Spence M.S. (2019). Eosinopenia as an Adverse Marker of Clinical Outcomes in Patients Presenting with Acute Myocardial Infarction. Am. J. Med..

[48] Akbar N., Braithwaite A.T., Corr E.M., Koelwyn G.J., van Solingen C., Cochain C., Saliba A.E., Corbin A., Pezzolla D., Moller J.M. (2022). Rapid neutrophil mobilisation by VCAM-1+ endothelial extracellular vesicles. Cardiovasc Res..

[49] Alkhalil M., Edmond E., Edgar L., Digby J.E., Omar O., Robson M.D., Choudhury R.P. (2018). The relationship of perivascular adipose tissue and atherosclerosis in the aorta and carotid arteries, determined by magnetic resonance imaging. Diabetes Vasc. Dis. Res..

[50] Oikonomou E.K., Marwan M., Desai M.Y., Mancio J., Alashi A., Hutt C.E., Thomas S., Herdman L., Kotanidis C.P., Thomas K.E. (2018). Non-invasive detection of coronary inflammation using computed tomography and prediction of residual cardiovascular risk (the CRISP CT study): A post-hoc analysis of prospective outcome data. Lancet.

[51] Ridker P.M., Cushman M., Stampfer M.J., Tracy R.P., Hennekens C.H. (1997). Inflammation, Aspirin, and the Risk of Cardiovascular Disease in Apparently Healthy Men. N. Engl. J. Med..

[52] Ridker P.M., Danielson E., Fonseca F.A., Genest J., Gotto A.M., Kastelein J.J., Koenig W., Libby P., Lorenzatti A.J., MacFadyen J.G. (2009). Reduction in C-reactive protein and LDL cholesterol and cardiovascular event rates after initiation of rosuvastatin: A prospective study of the JUPITER trial. Lancet.

[53] Ridker P.M., Everett B.M., Thuren T., MacFadyen J.G., Chang W.H., Ballantyne C., Fonseca F., Nicolau J., Koenig W., Anker S.D. (2017). Antiinflammatory Therapy with Canakinumab for Atherosclerotic Disease. N. Engl. J. Med..

[54] Nidorf S.M., Fiolet A.T.L., Mosterd A., Eikelboom J.W., Schut A., Opstal T.S.J., Xu X.F., Lenderink T., Latchem D., Hoogslag P. (2020). Colchicine in Patients with Chronic Coronary Disease. N. Engl. J. Med..

[55] Tardif J.-C., Kouz S., Waters D.D., Bertrand O.F., Diaz R., Maggioni A.P., Pinto F.J., Ibrahim R., Gamra H., Kiwan G.S. (2019). Efficacy and Safety of Low-Dose Colchicine after Myocardial Infarction. N. Engl. J. Med..

[56] Kuzemczak M., Ibrahem A., Alkhalil M. (2021). Colchicine in Patients with Coronary Artery Disease with or Without Diabetes Mellitus: A Meta-analysis of Randomized Clinical Trials. Clin. Drug Investig..

[57] Edgar L., Akbar N., Braithwaite A.T., Krausgruber T., Gallart-Ayala H., Bailey J., Corbin A.L., Khoyratty T.E., Chai J.T., Alkhalil M. (2021). Hyperglycemia Induces Trained Immunity in Macrophages and Their Precursors and Promotes Atherosclerosis. Circulation.

[58] Ridker P.M., Rane M. (2021). Interleukin-6 Signaling and Anti-Interleukin-6 Therapeutics in Cardiovascular Disease. Circ. Res..

[59] Alkhalil M. (2019). Proprotein Convertase Subtilisin/Kexin Type 9 (PCSK9) Inhibitors, Reality or Dream in Managing Patients with Cardiovascular Disease. Curr. Drug Metab..

[60] O’Donoghue M.L., Murphy S.A., Sabatine M.S. (2020). The Safety and Efficacy of Aspirin Discontinuation on a Background of a P2Y(12) Inhibitor in Patients After Percutaneous Coronary Intervention: A Systematic Review and Meta-Analysis. Circulation.

[61] Galli M., Benenati S., Capodanno D., Franchi F., Rollini F., D’Amario D., Porto I., Angiolillo D.J. (2021). Guided versus standard antiplatelet therapy in patients undergoing percutaneous coronary intervention: A systematic review and meta-analysis. Lancet.

[62] Costa F., Van Klaveren D., Feres F., James S., Räber L., Pilgrim T., Hong M.-K., Kim H.-S., Colombo A., Steg P.G. (2019). Dual Antiplatelet Therapy Duration Based on Ischemic and Bleeding Risks After Coronary Stenting. J. Am. Coll. Cardiol..

[63] Gimbel M., Qaderdan K., Willemsen L., Hermanides R., Bergmeijer T., de Vrey E., Heestermans T., Gin M.T.J., Waalewijn R., Hofma S. (2020). Clopidogrel versus ticagrelor or prasugrel in patients aged 70 years or older with non-ST-elevation acute coronary syndrome (POPular AGE): The randomised, open-label, non-inferiority trial. Lancet.

[64] Ponchia P.I., Ahmed R., Farag M., Alkhalil M. (2022). Antiplatelet Therapy in End-stage Renal Disease Patients on Maintenance Dialysis: A State-of-the-art Review. Cardiovasc. Drugs Ther..

[65] Alkhalil M., Chai J.T., Choudhury R.P. (2017). Plaque imaging to refine indications for emerging lipid-lowering drugs. Eur. Heart J. Cardiovasc. Pharmacother..

[66] Alkhalil M., Biasiolli L., Chai J.T., Galassi F., Li L., Darby C., Halliday A., Hands L., Magee T., Perkins J. (2017). Quantification of carotid plaque lipid content with magnetic resonance T2 mapping in patients undergoing carotid endarterectomy. PLoS ONE.

[67] Alkhalil M., Biasiolli L., Akbar N., Galassi F., Chai J.T., Robson M.D., Choudhury R.P. (2018). T2 mapping MRI technique quantifies carotid plaque lipid, and its depletion after statin initiation, following acute myocardial infarction. Atherosclerosis.

[68] Chai J.T., Biasiolli L., Li L., Alkhalil M., Galassi F., Darby C., Halliday A.W., Hands L., Magee T., Perkins J. (2017). Quantification of Lipid-Rich Core in Carotid Atherosclerosis Using Magnetic Resonance T(2) Mapping: Relation to Clinical Presentation. JACC Cardiovasc. Imaging.

[69] Alkhalil M., Kuzemczak M., Whitehead N., Kavvouras C., Džavík V. (2021). Meta-Analysis of Intensive Lipid-Lowering Therapy in Patients with Polyvascular Disease. J. Am. Heart Assoc..

[70] Alkhalil M. (2020). Effects of intensive lipid-lowering therapy on mortality after coronary bypass surgery: A meta-analysis of 7 randomised trials. Atherosclerosis.

